# Evaluation of Polybutylate-Coated Braided Polyester (Ethibond) Sutures for Levator-Advancement Blepharoptosis Repair

**DOI:** 10.5402/2012/763731

**Published:** 2012-09-18

**Authors:** Michael Yulish, Joseph Pikkel

**Affiliations:** Ziv Medical Center, Ophthalmology Department, Safed 13100, Israel

## Abstract

*Purpose.* To evaluate the efficacy and safety of polybutylate-coated braided polyester (Ethibond* 5-0) suture for levator aponeurosis suturing to the anterior tarsal surface in involutional blepharoptosis repair surgery. *Methods.* Ten consecutive patients (16 eyes) with acquired blepharoptosis which resulted from levator aponeurosis dehiscence with good levator function had gone through surgery and were followed up for, at least, one year. *Results.* There was no significant change between postoperative MRD1 measurements. No serious complications, such as infection of the sutures, inflammation, granuloma formation or ptosis recurrence, were registered. *Conclusion.* Polybutylate-coated braided polyester (Ethibond* 5-0) suture is a safe and effective material for involutional blepharoptosis repair surgery.

## 1. Introduction

Involutional blepharoptosis occurs in the elderly population as a result of levator aponeurosis dehiscence from the anterior tarsal surface, which stretches or thins the aponeurotic fibers, and results in a lowered upper eyelid position and obstruction of the superior visual field [1–3]. Clinically, the patient presents with a lower upper eyelid position, superior migration of the upper eyelid crease and normal levator muscle excursion. Recruitment of the frontalis muscle to raise the eyebrow and compensate for loss of the superior visual field sometimes occurs, and contralateral eyelid retraction may be apparent in cases of unilateral or asymmetric ptosis.

Surgical repair is usually required to reattach the levator aponeurosis to the anterior face of the tarsal plate.One to three sutures are necessary. Suture materials that are used for this purpose include 6-0 silk, 6-0 or 5-0 polypropylene (Prolene*), 6-0 or 5-0 polyglactin, and 910 (Vicryl*). Each material has advantages as well as disadvantages for ptosis surgery.

The purpose of this study is to evaluate the efficacy and safety of polybutylate-coated braided polyester (Ethibond* 5-0; 8.0 mm, 1/4 c, spatula, Ethicon*, Somerville, NJ, USA) for suturing the levator aponeurosis to the anterior tarsal surface in involutional blepharoptosis repair surgery.

Ethibond is a very soft and easy to use multifilament suture. Ethibond knots are less bulky than knots of other suture materials [4]. The use of the nonabsorbable Ethibond reduces the risk of knots releasing as well as of ptosis recurrence. However, the nonabsorbable and multifilament structure of Ethibond has been reported to lead to infection, inflammation, or granuloma formation [5–8].

## 2. Methods

Ten consecutive patients with acquired blepharoptosis resulting from levator aponeurosis dehiscence were treated from May 2008 until August 2009 at the Oculoplastic Service in the Ziv Medical Center, Zefad, Israel. Eyelid ptosis was bilateral in six patients and unilateral in four. All underwent levator aponeurosis advancement technique ptosis repair. Exclusion criteria were Horner's syndrome, congenital ptosis, history of trauma, prior eyelid surgery, Grave's ophthalmopathy, Bell's palsy, treatment for glaucoma with topical medication, and concomitant blepharoplasty. Levator function (eyelid excursion at brow fixation) was at least 11-12 mm in all the patients. Margin-reflex distance (MRD1) (the distance between the upper eyelid margin and the corneal light reflex) was measured in the upright position by the surgeon with a handheld ruler, preoperative, and 1 week, 3 months, 6 months, 1 year and more than 1 year (in four patients) postoperatively. Complications, including ptosis recurrence, infection, inflammation, and granuloma formation, were recorded.

### 2.1. Surgical Technique

All procedures were performed under local anaesthesia. Patients were sedated by oral Diazepam five or ten mg. The skin crease was marked to be symmetric with that on the opposite side of the eyelid. Anaesthetic solution (2% lidocaine with 1 : 100 000 epinephrine) was injected subcutaneously into the eyelid. The incision was made through the skin to expose underlying orbicularis muscle; the wound was deepened through the orbicularis until the tarsal plate was exposed throughout the length of the incision. The dissection was directed upward posterior to the orbicularis muscle in order to expose the orbital septum. The septum was incised and the preaponeurotic fat pad and levator aponeurosis were exposed. The attenuated aponeurosis directly superior to the tarsal plate was incised and the edge of white and healthy aponeurosis was sutured by two or three Ethibond* 5-0 sutures to the tarsal plate. The sutures were adjusted according to eyelid position, in the supine position. The skin incision was sutured with polypropylene 6-0 sutures. An antibiotic ointment was prescribed to be applied to the incision twice daily during the first ten days. All the surgeries were performed by the same surgeon. 

Correlations between variables were calculated with Microsoft Excel (Microsoft Corporation, Redmond, Washington, DC, USA).

## 3. Results

Six of the patients (10 eyes, 62.5%) were women and four (6 eyes, 37.5%) were men. Patients' age ranged from 38 to 81 years; mean age was 67.8 years (Table 1). Ptosis was repaired in nine right eyelids (56%) and seven left eyelids (44%). Skin Polypropylene 6-0 suture infection was observed in two patients (patients 2 and 8, Table 1). They were treated by local antibiotic ointment and the infected sutures removed. Other complications, such as infection of polybutylate-coated braided polyester sutures, inflammation, granuloma formation or ptosis recurrence, were not observed.

**Figure 1:**
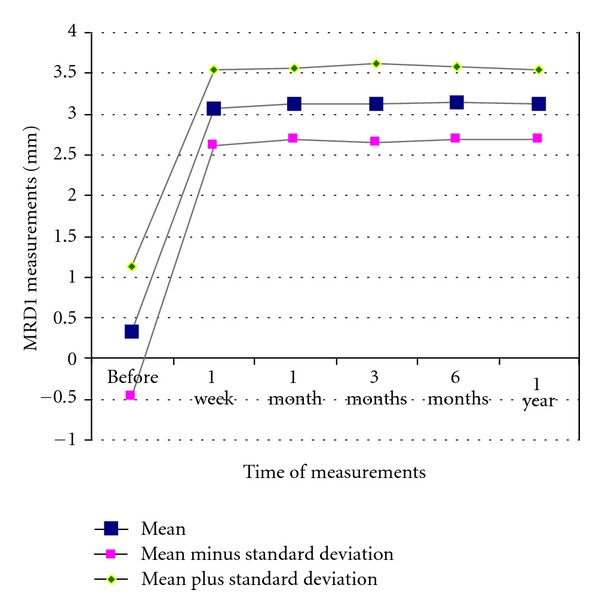
Changes in MRD1 measurements before and after surgery. *y*-axis: MRD1 measurements (mm). *x*-axis: Time of measurements.

Preoperative and postoperative MRD1 measurements are presented in Table 1. Mean values, standard deviations, and ranges are presented in Table 2. Mean MRD1 measurements at one week postoperatively were significantly higher than at preoperatively (Table 2, Figure 1). Mean MRD1 measurements did not change significantly during the one-year, or longer, follow-up period.

## 4. Discussion

We did not observe any serious complications, such as infection, inflammation, or granuloma formation following suturing of the anterior tarsal surface in involutional blepharoptosis repair surgery using polybutylate-coated braided polyester Ethibond 5-0. Skin polypropylene 6-0 suture infections appeared in two patients. No incidences of ptosis recurrence were observed during one-year followup.

As a nonabsorbable suture material, Ethibond, like silk, decreases the chance of ptosis recurrence due to knot release. Bartley et al. [9] used 6-0 silk sutures for levator-advancement ptosis repair, with successful results. However, silk is subject to gradual degradation and loss of strength, though this process may take several years. Many surgeons find silk the easiest of all suture materials to work with. On the other hand, silk, as a natural braided suture, causes inflammation and provides spaces for bacterial growth. Significant erythema around sutures, epithelial-lined suture tracks and suture abscesses may occur with the use of silk.

Another synthetic nonabsorbable suture material is polypropylene (Prolene*). Many surgeons use polypropylene 5-0 or 6-0 for aponeurosis suturing to the anterior tarsal surface. However, polypropylene can serve as a nidus for infection and has some stiffness, which can lead to early or late skin or conjunctiva extrusion of the suture's ends, and may be disturbing to patients. 

Polyglactin 910 [10] is a synthetic absorbable multifilament (braided) suture that is used to secure levator aponeurosis to the tarsus. The sutures are coated, reducing friction, enabling smooth and easy tissue passage. However, the sutures weaken within a few weeks, which may explain the risk of ptosis recurrence. The common believe is that the surgical scar is strong enough 1-2 weeks after the operation. Absorbable sutures create more inflammation than nonabsorbable ones and can lead to granuloma formation. However, they are less prone to serve as a nidus for infection than permanent sutures.

Ethibond has demonstrated efficacy as a suture material in other medical procedures. With its high tensile strength, Ethibond 5-0 has been shown to provide adequate fixation of the tibial tubercle osteotomy segment in revision knee arthroplasty, with reduced risk of complication as compared to conventional fixation methods that use screws and wires [11].

The sample size of the current study (10 patients, 16 eyes) is relatively small. In another study, Ethibond 4-0 sutures were used as sling material for frontalis suspension, of bilateral sutures granuloma in 15 patients (30 eyes). One patient required a granuloma excision, and bilateral suture abscesses in another patient prompted removal of sutures. In a third patient unilateral slippage of the knot occurred, with reappearance of ptosis in that eye. 

Ptosis recurrence was not marked in our patients, during the follow-up period, which was of at least one-year duration, and longer in four patients (7 eyes). Mean MRD1 increased from 0.33 mm preoperatively to 3.08 mm at 1 week postoperatively (*P* < 0.001), and remained steady from that point to the end of the follow-up period.

We found polybutylate-coated braided polyester (Ethibond* 5-0) suture to be a safe and effective material for levator aponeurosis suturing to the anterior tarsal surface in involutional blepharoptosis repair surgery.

## Figures and Tables

**Table 1:** MRD1 in ten patients (16 eyelids) with blepharoptosis.

					Postoperative MRD						
	Age	Gender	Eyelid	Preop. MRD	MRD	MRD	MRD	MRD	MRD	MRD	Complications
					1 week	1 month	3 months	6 months	1 year	>1 year	
1	64	F	R	0.5	2.9	3.0	3.0	3.0	3.0		
2	69	F	R	1.0	3.1	3.5	3.4	3.5	3.5	3.4	**, ^#^
3	38	M	L	1.5	4.0	3.8	4.0	4.0	3.8		
4	61	M	R	0	3.5	3.8	4.0	3.8	3.8		
5	70	F	R	0.5	3.0	2.9	2.8	2.9	2.9	2.8	
			L	1.0	2.8	2.8	2.8	2.9	2.9	2.8	
6	75	M	R	−1.0	2.5	2.7	2.8	2.7	2.7		
			L	0	3.0	3.1	3.0	2.9	3.0		
7	79	F	R	0.8	2.6	2.7	2.7	2.8	2.7		
			L	0	2.5	2.6	2.6	2.6	2.6		
8	81	F	R	−0.5	3.0	3.0	3.0	3.0	3.0	3.0	**, ^#^
			L	0.5	3.2	3.0	3.0	3.0	3.0	3.0	
9	63	F	R	1.0	2.9	2.8	2.8	2.9	2.9		
			L	1.5	2.7	2.8	2.7	2.8	2.8		
10	78	M	R	−1.0	4.0	3.9	3.9	3.9	3.9	3.8	
			L	−0.5	3.5	3.5	3.6	3.5	3.5	3.5	

Complications: *recurrence, **infection, ^#^inflammation, ^##^granuloma formation.

**Table 2:** Preoperative and postoperative mean MRD1 (±SD) in ten patients (16 eyelids).

	Preoperative	Postoperative				
		1 week	1 month	3 months	6 months	1 year
Mean MRD1 (SD) mm	0.33 (0.80)	3.08 (0.47)	3.12 (0.44)	3.13 (0.49)	3.14 (0.45)	3.13 (0.43)
Range, mm	−1.00 to 1.50	2.50 to 4.00	2.60 to 3.90	2.60 to 4.00	2.60 to 4.00	2.60 to 3.90
